# The Treatment of Cholera

**Published:** 1910-09-24

**Authors:** 


### THE TREATMENT OF CHOLERA.

### SOME NOTES ON DRUGS AND GENERAL MEASURES.

The fact that half of the patients who suffer from cholera recover with or without treatment indicates that the body tends to produce its own anti-substances, so that it is unwise to be too active in the administration of drugs or other remedies. From the public point of view the first essential is prophylaxis, and this depends upon the early recognition of the first case, its isolation, and attention to the source from which the bacteria were acquired in that case, followed by the destruction of any bacteria that may remain at that source. Anti-cholera vaccination has been employed extensively, and there is evidence to show that it is beneficial and protective when the inoculations have been recent, though these effects last but a comparatively short time. The chief principles of treatment of an individual case consist in, first, checking the preliminary diarrhœa; this may be accomplished by the use of astringents with or without opium, a familiar Indian cholera pill consisting of opium, asafœtida, and black pepper. Acetate of lead may be given with opium; vegetable astringents such as krameria, kino, hæmotoxylum, ipecacuanha, tannic acid, gallic acid, may also be resorted to, and chlorodyne may be serviceable in relieving any griping. The precise dose of the remedies employed and the intervals at which the draught or pill should be repeated must necessarily vary with the age of the patient, the severity of the attack, and the experience of the physician, but upon the whole a fairly large dose at fairly frequent intervals may be employed. If the stomach will retain nothing, hypodermic injections of morphia become necessary. Another line of treatment having the same end in view is the application of a mustard leaf to the epigastric angle, or the use of a general poultice to the abdomen.

#### Rest and Nourishment.

It is clear that the patient needs complete rest in bed, and he should be left quiet and to himself as much as possible. Sleep should on no account be interrupted. The extremities will need keeping warm with hot bottles or hot blankets, and to prevent the great depression of the surface temperature which takes place in the collapse stage of cholera it is important that the temperature of the room should not be allowed to fall below 70° F., though ventilation should still be free, and the patient should not be weighted down with too many blankets. Thirst is a very marked symptom, and it needs continuous treatment by the exhibition of small quantities of iced-water or milk and soda, small lumps of ice to suck, rinsing of the mouth or lips with a weak solution of lemon juice; large draughts should be avoided, for they accentuate the vomiting. Thirst may be lessened, and collapse at the same time guarded against, by the infusion of normal saline fluid at body temperature into the subcutaneous tissues by the continuous method; it is not possible to supply fluid per rectum on account of the rapidly repeated evacuations.

The dieting of cholera is a very difficult task, owing to the persistence of both sickness and diarrhœa; during the acute stage it may be impossible for the patient to retain anything. Fortunately in the less unfavourable cases the vomiting may cease of itself within forty-eight hours, and then small feeds of the simplest fluids, particularly milk or preparations of milk, may be taken, and increased in amount when the patient shows that he can retain and digest them.

#### Collapse and Heart Failure.

Failure of the heart in the stage of collapse requires very careful treatment indeed. Brandy may be given by the mouth, or iced champagne if the stomach will retain them, in teaspoonful doses, and this may be all that is required; more often, however, hypodermic injections of ether, strychnine, or brandy may be required to carry the patient through the crisis; oxygen inhalations given for five minutes at a time every half-hour often serve as a very good cardiac stimulant; the foot of the bed may be raised and the legs bandaged, whilst continuous infusion with normal saline solution at rather over body temperature, into the subcutaneous tissues of the breasts or axillæ with all aseptic precautions will generally be the most efficient remedy for the heart in cases of severe collapse. All measures should be taken with extreme gentleness, the patient being disturbed as little as possible. It is probable that the addition to the infused fluid of very small doses (*e.g.*, 1 cc. of 20 per cent. solution) of pituitary extract would have a beneficial effect here, as it does in cases of surgical collapse, but this line of treatment has not been extensively employed in cholera yet.

